# Association between tobacco and/or alcohol consumption during pregnancy and infant development: BRISA Cohort

**DOI:** 10.1590/1414-431X202010252

**Published:** 2020-12-21

**Authors:** M.E.A. Negrão, P.R.H. Rocha, M.C.P. Saraiva, M.A. Barbieri, V.M.F. Simões, R.F.L. Batista, A.A. Ferraro, H. Bettiol

**Affiliations:** 1 Departamento de Puericultura e Pediatria, Faculdade de Medicina de Ribeirão Preto, Universidade de São Paulo, Ribeirão Preto, SP, Brasil; 2 Departamento de Clínica Infantil, Faculdade de Odontologia Ribeirão Preto, Universidade de São Paulo, Ribeirão Preto, SP, Brasil; 3 Departamento de Saúde Pública, Universidade Federal do Maranhão, São Luís, MA, Brasil; 4 Departamento de Pediatria, Faculdade de Medicina, Universidade de São Paulo, São Paulo, SP, Brasil

**Keywords:** Motor skills, Cognition, Cohort studies, Risk factors, Prenatal exposure, Delayed effects

## Abstract

Fetuses exposed to alcohol and/or tobacco are at risk for perinatal adversities. However, little is currently known about the association of the separate or concomitant use of alcohol and tobacco with infant motor and cognitive development. Thus, the objective of the present study was to investigate the association between maternal consumption of alcohol and/or tobacco during pregnancy and the motor and cognitive development of children starting from the second year of life. The study included 1006 children of a cohort started during the prenatal period (22-25 weeks of pregnancy), evaluated at birth and reevaluated during the second year of life in 2011/2013. The children were divided into four groups according to the alcohol and/or tobacco consumption reported by their mothers at childbirth: no consumption (NC), separate alcohol consumption (AC), separate tobacco consumption (TC), and concomitant use of both (ACTC). The Bayley Scale of Infant and Toddler Development Third Edition screening tool was used for the assessment of motor and cognitive development. Adjusted Poisson regression models were used to determine the association between groups and delayed development. The results indicated that only the ACTC group showed a higher risk of motor delay, specifically regarding fine motor skills, compared to the NC group (RR=2.81; 95%CI: 1.65; 4.77). Separate alcohol or tobacco consumption was not associated with delayed gross motor or cognitive development. However, the concomitant use of the two substances increased the risk of delayed acquisition of fine motor skills.

## Introduction

Habits such as alcohol and/or tobacco consumption during the gestational period are risk factors for both maternal and fetal health (1,2). During pregnancy, ingested alcohol diffuses through body tissues, fluids, and the placenta, reaching the amniotic fluid. As a consequence of this process, subtle or even severe modifications may occur during the course of fetal growth and the development of the central nervous system (3,4). In turn, toxins from cigarettes such as carbon monoxide, nicotine, cyanide, cadmium, and lead may cause changes in placental function, which then reduce the oxygen and nutrient supply for the fetus, increasing the risk of perinatal adversities (5,6). Nicotine also acts as a neurological teratogen as it crosses over the placental barrier and can trigger nicotine receptors of acetylcholine, altering the development of nervous tissues. Thus, as shown in different studies, fetuses that are exposed to nicotine can present a deficit in the number of neurons and important alterations in sensorial-cognitive functions (7–9).

In addition to perinatal health problems, fetal exposure to alcohol and/or tobacco may be related to damage to different developmental parameters over time (10). The effect of alcohol on infant development may vary according to the frequency, quantity, and period of fetal exposure to the substance (11,12). Bandoli et al. (11) reported that continuous consumption of high alcohol doses throughout gestation was associated with low mental and psychomotor performance of the offspring during the first year of life. Conversely, low-moderate alcohol consumption interrupted at the beginning of gestation was not associated with delayed infant development. Regarding tobacco consumption during pregnancy, although some studies have suggested an association with damage to developmental and behavioral parameters (6,13), investigations taking into consideration confounding factors such as family socioeconomic variables and maternal mental health have not indicated an association between smoking during pregnancy and behavioral delays during infancy (13–17).

Although several studies have investigated the effect of alcohol or tobacco consumption during pregnancy on motor and cognitive behavior, in general they have not considered the fetuses exposed to both substances. Investigations regarding perinatal outcomes have indicated that fetuses concomitantly exposed to alcohol and tobacco are at higher risk for prematurity and intrauterine growth restriction compared to peers exposed to only one or none of these substances (19,20). However, little is currently known about the effect of the concomitant use of alcohol and tobacco during pregnancy on infant motor and cognitive outcomes compared to groups exposed to only one or none of these substances. Thus, the objective of the present study was to investigate the association between maternal consumption of alcohol and/or tobacco during pregnancy and the motor and cognitive development of children starting from the second year of life.

## Material and Methods

### Participants

This was a prospective cohort study conducted on a convenience sample of pregnant women starting during the prenatal period (2010). Mothers and children were evaluated at birth (2010/2011) and from the beginning of the second year of life (2011/2013). The data of this study are part of an investigation entitled “Etiological factors of preterm birth and consequences of perinatal factors on children's health: birth cohorts of two Brazilian cities - BRISA (acronym of “Brazilian Birth Cohort Studies, Ribeirão Preto and São Luís”) (21). The objective of the BRISA study was to investigate factors associated with preterm birth and the repercussions of prematurity and of other early events on lifelong health. Data for the Ribeirão Preto (RP) cohort were analyzed in the present study. In 2010, the city had a population of 604,000, with a Human Development Index (HDI) of 0.80, occupying the 40th position in the HDI ranking of 5565 Brazilian cities (22).

A convenience sample was used because of the impossibility of obtaining a representative random sample of pregnant women in this population due to the lack of records of pregnant women or of women who received prenatal care. Pregnant women evaluated in public and private services were invited to participate in the prenatal BRISA cohort according to the following inclusion criteria: having had an obstetric ultrasound exam before the 20th week of pregnancy, having a gestational age of 22 to 25 weeks at the time of data collection, and carrying a singleton pregnancy. Thus, the prenatal RP cohort included 1400 pregnant women and data were collected from February 2010 to February 2011. By conducting interviews and using a standardized questionnaire, a previously trained team obtained data about reproductive health, demographic and socioeconomic data, characteristics of pregnancy, depressive symptoms, and life habits of the selected subjects. Subjects were interviewed and evaluated at the Clinical Research Unit of the University Hospital, Ribeirão Preto Medical School, University of São Paulo (HCFMRP-USP).

From April 2010 to June 2011, 97.8% (n=1369) of the women from the original cohort and their newborns participated in the study at the time of birth. The losses in relation to the initial sample were due to one case of abortion, and 30 other cases due to refusal to do the interview after delivery, failure to locate the mother during hospitalization, and early discharge from the hospital. Teams of interviewers trained by the investigators visited the maternity hospitals daily in order to conduct standardized questionnaires with the puerperae. The following data were collected through interviews with the mothers in the maternity hospitals: identification, reproductive health data, characteristics of the pregnancy, delivery and birth, maternal characteristics and life habits, including smoking and consumption of alcoholic beverages during pregnancy, demographic and social information, and health problems during childbirth. Newborn anthropometry data (weight, length, and head circumference) were collected by the team from patient records at the hospitals.

**Figure 1:**
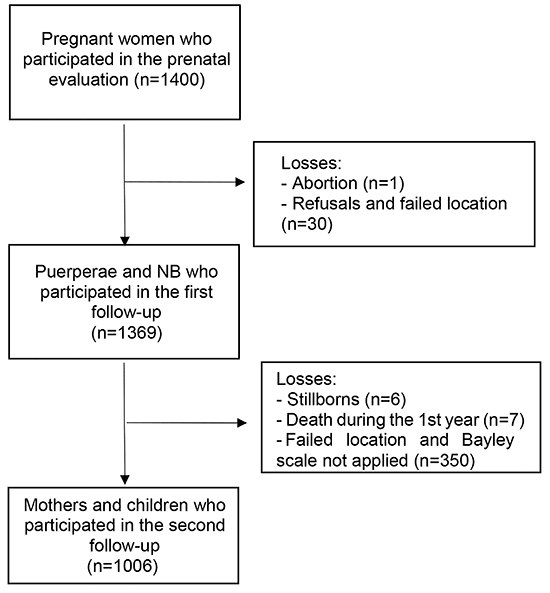
Flow diagram of the participants in the prenatal BRISA cohort in Ribeirão Preto, SP, Brazil. NB: newborn.

A follow-up study with mothers and children who participated in the previous stages of the study was conducted starting in the second year of child's life. In this phase, the children's development was evaluated by previously trained psychologists. The assessment was performed in an appropriate room at HCFMRP-USP. During the first year of life, the teams identified six cases of stillbirths and seven deaths, besides the abortion already identified in the previous phase. Thus, 1,356 participants were eligible for evaluation, with the participation of 1,081 mothers and children in the follow-up. Specifically in the present study, 75 participants who did not perform the evaluation were excluded, resulting in 1,006 participating children (74.2% of the eligible). None of the children from the follow-up stage had congenital or acquired health problems that would justify their exclusion from the study (Figure 1). All the procedures of the present study were approved by the Research Ethics Committee of HCFMRP-USP (protocol No. 11157/2008).

### Independent variable

The independent variable was the combination of alcohol and tobacco consumption reported by the mother during pregnancy when interviewed at the moment of childbirth. The following questions were asked: “Did you drink beer during pregnancy?”; “Did you drink wine during pregnancy?”; “During pregnancy, did you have any other type of drink such as whiskey, vodka, gin or rum?”; “Did you smoke during this pregnancy?” The participant was considered a smoker when she reported smoking any number of cigarettes per day; alcohol consumption was considered present when she reported the intake of any amount of at least one type of the above alcoholic beverages during pregnancy. On this basis, this variable consisted of four categories: no consumption (NC), only alcohol consumption (AC), only tobacco consumption (TC), and both alcohol and tobacco consumption (ACTC).

Although not taken into account in the definition of the independent variable, the level of alcohol exposure during the gestational trimesters was calculated in order to better characterize the consumption profile for the AC and ACTC groups. When the pregnant women reported having consumed any type of alcohol beverage, as shown previously, they were asked in which gestational period, the frequency, and intensity of consumption. According to this information, alcohol consumption during pregnancy was classified as: low (1 to 20 g absolute alcohol per day), moderate (21 to 40 g absolute alcohol per day), or high (41 g or more absolute alcohol) based on the percentage of absolute alcohol present in each drink (5% in beer, 12% in wine, and 40% in liquors) (23). Regarding smoking, in order to identify tobacco use profile, tobacco smoking by the TC and ACTC groups was classified as ≤5 cigarettes per day, 6 to 10 cigarettes per day, and >10 cigarettes per day. The gestational period of the smoking was not specified.

### Dependent variable

The following instruments were used to assess motor and cognitive development: the fine motor subscale (FMS), the gross motor subscale (GMS), and the cognitive scale (CS) of the Bayley Scale of Infant and Toddler Development Third Edition - screening (Bayley-III screening) (24). The infants were evaluated within the 13-to-30-month age range during cohort follow-up. This scale permitted us to determine if development was progressing according to normal expectations or if a more in-depth assessment was necessary. The FMS instrument contains tests that permit the determination of handgrip, perceptive-motor integration, and motor planning, and the GMS assesses tasks involving interlimb coordination, displacement, motor planning, and postural stability. The cognitive subscale of the instrument includes tasks for the assessment of attention, preferences regarding novelty and habituation, problem solving, exploration and manipulation, concept formation, and other aspects of cognitive development.

Ten psychologists were trained for the administration of Bayley's test by a psychologist with experience in the use of the instrument. Training occurred in groups and started with expository lessons on theoretical concepts behind the instrument, its psychometric qualities, forms of administration, and analysis. Afterwards, the psychologists observed three hours of administration of the test with volunteers in order to discuss possible difficulties in the administration and/or questions regarding the scores. Subsequently, the professionals applied the instrument with the supervision of the psychologist. After three supervised administrations of the test, they started conducting it on their own.

For evaluation, the age of preterm infants was corrected by subtracting from the chronological age of follow-up the number of weeks up to the gestational age of 40 weeks. Performance on the subscales was analyzed based on the cut-off point for age established by the scale itself as Competent, Emergent, and at Risk. In the present study, the classifications were analyzed dichotomously as competent and emergent/at risk.

### Confounding variables

A directed acyclic graph (DAG) was constructed for the identification of confounding variables using the DAGitty software version 2.3 (http://dagitty.net/). The DAG is a causal diagram constructed based on known theoretical assumptions about certain causal relations. Based on the heuristic rules of the constructed diagram, it is possible to identify potential confounding variables for adjustment of the analytical model proposed.

Thus, the following variables obtained during the prenatal phase were considered to be potentially confounders: mother's schooling as years of study (categorized into >12, 9-11, and ≤8 years of study), mother's age (categorized into <20 years, 20-34 years, and >34 years), economic classes according to the Brazilian Economic Classification Criterion of the Brazilian Association of Research Enterprises (ABEP) (25) (categorized into A/B, C, and D/E, with A/B being the more privileged and D/E the more underprivileged), mother's marital situation (married, consensual union, or no partner), and depressive symptoms determined with the Center for Epidemiological Studies - Depression Scale (CES-D) (26). The cut-off point for the presence of depressive symptoms was ≥24 points on the scale. The remaining variables presented in Supplementary Figure S1 were not identified as confounding variables and, therefore, were not considered for analysis.

However, for a better characterization of the sample, we identified prematurity and intrauterine growth restriction, although they were not considered confounding variables by DAG for the analysis. For classification, we used the birth weight ratio, defined as the ratio between birth weight and the mean weight for sex and gestational age based on the curve of the International Fetal and Newborn Growth Consortium for the 21st Century (INTERGROWTH21st) (27). A birth weight ratio <0.85 was defined as intrauterine growth restriction (28). In order to identify pre-term birth (gestational age <37 weeks), the gestational age was calculated using two pieces of information: the ultrasound and the last menstrual period reported by the mother during the prenatal interview. When these two pieces of information were compatible, considering an error of ±7% for the ultrasound, only the date of the last menstrual period was considered; if incompatible, the ultrasound information was considered (29).

### Statistical analysis

The chi-squared test was used to compare the characteristics of subjects absent and present during follow-up to those of the groups (NC, AC, TC, ACTC). The relationship between groups and classifications on the Bayley III subscale was determined by calculating the relative risk using a Poisson regression method with robust estimate of variance and with adjustment for the covariates identified by the DAG. The level of significance was set at 5% for all tests and the data were analyzed with the Stata statistical package, version 14 (USA).

## Results

Comparison of the characteristics of the participants absent and present during follow-up revealed differences regarding mother's age and economic class. A lower participation of mothers aged <20 years (14.2 *vs* 19.4%) and belonging to the D/E economic class (9.4 *vs* 16.9%) was observed during follow-up (Table 1).

**Table 1:** Characteristics of the participants who were present or absent during follow-up in Ribeirão Preto, SP, Brazil, 2010/13.

Characteristics	Total no. of participants n=1356	Absent during follow-up n=350	Present during follow-up n=1006	P value*
	n (%)	n (%)	n (%)	
Intrauterine growth restriction				0.251
No	1226 (90.4)	311 (88.9)	915 (90.9)	
Yes	130 (9.6)	39 (11.1)	91 (9.1)	
Gestational age (weeks)				0.915
≥37	1234 (91.0)	319 (91.1)	915 (90.9)	
<37	122 (9.0)	31 (8.9)	91 (9.1)	
Newborn's gender				0.990
Male	666 (49.1)	172 (49.0)	494 (49.1)	
Female	690 (50.9)	178 (51.0)	512 (50.9)	
CES-D				0.095
Without depressive symptoms	990 (77.3)	255 (80.7)	735 (76.2)	
With depressive symptoms	291 (22.7)	61 (19.3)	230 (23.8)	
Marital status				0.183
Married	509 (37.5)	117 (33.4)	392 (39.0)	
Consensual union	648 (47.8)	178 (50.9)	470 (46.7)	
No partner	199 (14.7)	55 (15.7)	144 (14.3)	
Mother's schooling (years)				0.079
≥12	109 (8.1)	29 (8.4)	80 (8.0)	
9-11	856 (63.3)	203 (58.5)	653 (65.0)	
≤8	387 (28.6)	115 (33.1)	272 (27.0)	
Mother's age (years)				0.004
20-34	1.017 (75.0)	255 (72.9)	762 (75.8)	
<20	211 (15.6)	68 (19.4)	143 (14.2)	
≥35	128 (9.4)	27 (7.7)	101 (10.0)	
Economic class				0.001
A/B	372 (27.9)	93 (27.2)	279 (28.1)	
C	810 (60.8)	191 (55.9)	619 (62.5)	
D/E	151 (11.3)	58 (16.9)	93 (9.4)	

The difference in the totals in relation to the reference number (n) are due to missing information. *Chi-squared test.

Regarding the comparison of groups present at follow-up, 68.9% of the subjects belonged to group NC, 18.6% to group AC, 6.3% to group TC, and 6.2% to group ACTC. The ACTC group showed a higher relative frequency of infants born with intrauterine growth restriction (17.7%) and a higher relative frequency of mothers with depressive symptoms (36.7%), and without a partner (32.2%) (Table 2).

**Table 2:** Comparison of the characteristics of groups NC, AC, TC, and ACTC in Ribeirão Preto, SP, Brazil, 2010/13.

Characteristics	Groups				P value*
	NC	AC	TC	ACTC	
	n (%)	n (%)	n (%)	n (%)	
	n=693 (68.9)	n=187 (18.6)	n=64 (6.3)	n=62 (6.2)	
Intrauterine growth restriction					0.016
No	638 (92.1)	172 (92.0)	54 (84.4)	51 (82.3)	
Yes	55 (7.9)	15 (8.0)	10 (15.6)	11 (17.7)	
Gestational age (weeks)					0.059
≥37	636 (91.8)	172 (92.0)	56 (87.5)	51 (82.3)	
<37	57 (8.2)	15 (8.0)	8 (12.5)	11 (17.7)	
Newborn's gender					0.782
Male	334 (48.2)	98 (52.4)	32 (50.0)	30 (48.4)	
Female	359 (51.8)	89 (47.6)	32 (50.0)	32 (51.6)	
CES-D					0.025
No depressive symptoms	519 (78.2)	136 (76.0)	42 (67.7)	38 (63.3)	
Depressive symptoms	145 (21.8)	43 (24.0)	20 (32.3)	22 (36.7)	
Marital situation					<0.001
Married	302 (43.6)	69 (36.9)	11 (17.2)	10 (16.1)	
Consensual union	308 (44.4)	91 (48.7)	39 (60.9)	32 (51.6)	
No partner	83 (12.0)	27 (14.4)	14 (21.9)	20 (32.3)	
Mother's schooling (years)					0.417
≥12	54 (7.8)	16 (8.6)	5 (7.8)	5 (8.1)	
9-11	457 (65.9)	124 (66.7)	34 (53.1)	38 (61.3)	
≤8	182 (26.3)	46 (24.7)	25 (39.1)	19 (30.6)	
Mother's age (years)					0.446
20-34	524 (75.6)	142 (75.9)	48 (75.0)	48 (77.4)	
<20	107 (15.4)	20 (10.7)	9 (14.1)	7 (11.3)	
≥35	62 (8.9)	25 (13.4)	7 (10.9)	7 (11.3)	
Economic class					0.375
A/B	184 (27.1)	54 (29.0)	16 (25.4)	25 (40.3)	
C	435 (64.0)	111 (59.7)	41 (65.1)	32 (51.6)	
D/E	61 (8.9)	21 (11.3)	6 (9.5)	5 (8.1)	
Age at follow-up (months)**					0.693
Mean (SD)	22.5 (3.3)	21.9 (2.8)	22.5 (3.8)	21.3 (2.9)	

The difference in the totals in relation to the reference number (n) were due to missing information. NC: no consumption; AC: only alcohol consumption; TC: only tobacco consumption; ACTC: alcohol and tobacco consumption. *Chi-squared test; **ANOVA.

Most of the pregnant women consumed a quantity of alcohol considered low (<20 g/day) during the three trimesters of gestation (Supplementary Table S1). Data also did not show differences in the levels of consumption during gestation in the ACTC and AC groups. In addition, among smokers, 76.2% in the TC group and 82.0% in the ACTC group reported smoking ≤10 cigarettes per day (Supplementary Table S2). There was no difference in the quantity of cigarettes by groups ACTC and TC**.**

Table 3 presents the distribution of the groups according to the developmental subscales. Differences between groups were observed only for FMS, with group ACTC showing a higher relative frequency of infants classified as emergent/at risk compared to the other groups (25.8%).

**Table 3:** Absolute and relative frequency of participants in the groups of alcohol and tobacco consumption and classification on the developmental subscales in Ribeirão Preto, SP, Brazil, 2010/13.

Classification on the subscale	Total	NC	AC	TC	ACTC	P value*
	n (%)	n (%)	n (%)	n (%)	n (%)	
FMS						<0.001
Competent	917 (91.2)	637 (91.9)	173 (92.5)	61 (95.3)	46 (74.2)	
Emergent/at risk	89 (8.8)	56 (8.1)	14 (7.5)	3 (4.7)	16 (25.8)	
GMS						0.221
Competent	906 (90.1)	628 (90.6)	171 (91.4)	54 (84.4)	53 (85.5)	
Emergent/at risk	100 (9.9)	65 (9.4)	16 (8.6)	10 (15.6)	9 (14.5)	
CS						0.841
Competent	861 (85.6)	595 (85.9)	159 (85.0)	56 (87.5)	51 (82.3)	
Emergent/at risk	145 (14.4)	98 (14.1)	28 (15.0)	8 (12.5)	11 (17.7)	

NC: no consumption; AC: only alcohol consumption; TC: only tobacco consumption; ACTC: alcohol and tobacco consumption; FMS: Fine motor subscale; GMS: Gross motor subscale; CS: Cognitive subscale. *Chi-squared test.

The adjusted Poisson regression model with robust estimate of variance revealed that the ACTC group had a higher risk of being classified as emergent/at risk on the FMS (RR=2.81, 95%CI: 1.65; 4.77, P<0.001) than the reference group (Table 4). However, on the remaining developmental subscales, no group showed higher risks than the NC group.

**Table 4:** Adjusted Poisson regression analysis of the association between groups of alcohol and tobacco consumption and the Bayley developmental subscales in Ribeirão Preto, SP, Brazil, 2010/13.

Group	FMS		GMS		CS	
	RR	95%CI	RR	95%CI	RR	95%CI
NC	1.00		1.00		1.00	
AC	0.91	0.51; 1.62	0.91	0.53; 1.55	0.97	0.65; 1.44
TC	0.56	0.18; 1.72	1.65	0.88; 3.10	0.77	0.39; 1.52
ACTC	2.81	1.65; 4.77	1.49	0.77; 2.87	1.04	0.58; 1.86

Model adjusted for maternal schooling and age, economic class, marital status, and depressive symptoms. NC: no consumption; AC: only alcohol consumption; TC: only tobacco consumption; ACTC: alcohol and tobacco consumption; FMS: Fine motor subscale; GMS: Gross motor subscale; CS: Cognitive subscale.

## Discussion

The present study investigated the association of maternal consumption of alcohol or tobacco, or both, during pregnancy with motor and cognitive development of the child during the second year of life. The results revealed risks of delay on the FMS in children concomitantly exposed to the two substances. Conversely, no difference was detected between the AC and TC groups compared to the reference group (NC).

The similarity of the AC group to the NC group may be explained by the fact that practically all participants reported low alcohol consumption during pregnancy. Halliday et al. (30) also did not observe a relationship between low/moderate maternal alcohol consumption and low performance of their offspring on the Bayley III scale at 24 months of age. A meta-analysis published by Flak et al. (12) did not detect an association between behavioral delays and exposure to a low/moderate quantity of alcohol during the prenatal period, but did reveal developmental losses in fetuses exposed to high doses. However, these results should be interpreted with caution since different parameters and criteria for the classification of the amount of alcohol consumption are used in the various studies. In addition, other indicators such as sustained consumption and period during which the fetus is exposed to alcohol could be risk factors for delayed infant development and should be considered (11).

The data of the present study did not reveal an association between separate tobacco consumption by pregnant women and delayed infant development. Several studies have shown that the relationship between maternal smoking habit and deficits of infant development may be attenuated by factors such as socioeconomic family situation, maternal education, domestic environment, psychiatric conditions of the parents, and infant birth conditions considered in the analyses (14–17). A population-based cohort study by Roza et al. (17) did not detect harmful effects of maternal smoking on child behavioral measurements at 18 months of life when the analysis was adjusted for family socioeconomic situation and parental mental health regardless of the number of cigarettes smoked. On the other hand, Huijbregts et al. (14) pointed out that the association of maternal tobacco consumption with motor and cognitive deficits during infancy is significantly mediated by newborn weight and is influenced by confounding factors such as family income and maternal education. In contrast, a systematic review published by Polanska et al. (31) pointed out that, even though the data regarding the relationship between delayed infant development and maternal tobacco consumption are inconsistent due to the low sensitivity of the tests in identifying delays during the first years of life, studies conducted on schoolchildren and adolescents consistently report cognitive impairment and low academic performance among children exposed to tobacco during the prenatal period (32).

Despite the similarities between the AC and TC groups regarding levels of alcohol and cigarette use, data indicate that the concomitant use of the substances is associated with delays in the FMS. In our study, the analyses were adjusted for education and mother's age, marital status, economic class, and mental health of the pregnant women. Nevertheless, the consumption of both substances turned out to be harmful for development. On the other hand, the lack of association with GMS and CS delays may be related to the non-linearity that often characterizes behavioral parameters. In contrast to motor deficits, which can be identified early, cognitive delays tend to appear during the acquisition of more complex cognitive skills over the years (18,33). The same is observed in children exposed to other perinatal risk factors. These groups often show recovery of GMS deficits by 12 months of age (34), delayed FMS during the second year of life (35), and lower-than-expected performance at school age (36).

Although it is difficult to distinguish the effect of each substance on the fetus, a few studies suggest that alcohol and tobacco act in a synergistic way, increasing risks for perinatal adversities (19,20). In our population, intrauterine growth restriction (IUGR) and prematurity were more frequent in the ACTC group than in others. Considering that prematurity and IUGR are associated with delays in the fine motor development during childhood (35,37), these conditions could be acting as mediators of the association between the concomitant use of alcohol and tobacco during gestation with delays in the development of fine motor skills, although this hypothesis was not tested in the present study.

We opted to use self-reporting intake as proxy for alcohol and tobacco consumption, which may have involved recall bias and omission of information due to social stigmas. However, this method permits the assessment of alcohol and tobacco consumption in studies that involve a large number of participants. Some particular characteristics of the present cohort, such as a sample consisting of women with a single fetus and with at least one prenatal visit during pregnancy, may differentiate it from the general population, whose consumption of the two substances could be higher.

The following strong points of the present study should be highlighted: i) it was a cohort investigation with three prospective measurements started during the prenatal period; ii) there was relatively high representativeness of follow-up in relation to the initial phase (74.2%); and iii) the study was original for investigating the motor and cognitive development of children concomitantly exposed to alcohol and tobacco during the prenatal period.

In conclusion, separate alcohol or tobacco consumption was not related to the risk of motor and cognitive delays during the second year of life. It is important to highlight that most participants reported low consumption of alcohol and tobacco. However, concomitant exposure to the two substances, although in low levels, increased the risk of delayed acquisition of fine motor skills. Prenatal care is an important tool to identify these risk factors early in pregnancy, so that preventive and treatment measures can be implemented.

## Supplementary Material

Click here to view [pdf].
